# Effects of Targeted Suppression of Glutaryl-CoA Dehydrogenase by Lentivirus-Mediated shRNA and Excessive Intake of Lysine on Apoptosis in Rat Striatal Neurons

**DOI:** 10.1371/journal.pone.0063084

**Published:** 2013-05-02

**Authors:** Jinzhi Gao, Cai Zhang, Xi Fu, Qin Yi, Fengyan Tian, Qin Ning, Xiaoping Luo

**Affiliations:** 1 Department of Pediatrics, Tongji Hospital, Tongji Medical College, Huazhong University of Science and Technology, Wuhan, China; 2 Department of Infectious Diseases, Tongji Hospital, Tongji Medical College, Huazhong University of Science and Technology, Wuhan, China; Universidade Federal do ABC, Brazil

## Abstract

In glutaric aciduria type 1 (GA1), glutaryl-CoA dehydrogenase (GCDH) deficiency has been shown to be responsible for the accumulation of glutaric acid and striatal degeneration. However, the mechanisms by which GA1 induces striatal degeneration remain unclear. In this study, we aimed to establish a novel neuronal model of GA1 and to investigate the effects of GCDH deficiency and lysine-related metabolites on the viability of rat striatal neurons. Thus we constructed a lentiviral vector containing short hairpin RNA targeted against the GCDH gene expression (lentivirus-shRNA) in neurons. A virus containing a scrambled short hairpin RNA construct served as a control. Addition of lysine (5 mmol/L) was used to mimic hypermetabolism. Cell viability was measured using 3-(4,5-dimethylthiazol-2-yl)-2,5-diphenyltetrazolium bromide. Apoptosis was assessed using Hoechst33342 staining and Annexin V-PE/7-AAD staining. The mitochondrial membrane potential (MPP) was monitored using tetramethylrhodamine methyl ester. The expression levels of caspases 3, 8, and 9 were determined by Western blotting. We found that lentivirus-shRNA induced apoptosis and decreased MMP levels in neurons, and addition of 5 mmol/L lysine enhanced this effect markedly. Lentivirus-shRNA upregulated the protein levels of caspases 3 and 9 regardless of the presence of 5 mmol/L lysine. The expression level of caspase 8 was higher in neurons co-treated with lentivirus-shRNA and 5 mmol/L lysine than in control. Benzyloxy-carbonyl-Val-Ala-Asp(OMe)-fluoromethylketone, a pan-caspase inhibitor, blocked the apoptosis induced by lentivirus-shRNA and 5 mmol/L lysine to a great extent. These results indicate that the targeted suppression of GCDH by lentivirus-mediated shRNA and excessive intake of lysine may be a useful cell model of GA1. These also suggest that GA1-induced striatal degeneration is partially caspase-dependent.

## Introduction

Glutaric aciduria type 1 (GA1) is an autosomal recessive inherited neurodegenerative disease caused by a deficiency in the activity of glutaryl-CoA dehydrogenase (GCDH). The overall prevalence is approximately 1 in 100,000 newborns, but this varies among different countries [1], [2]. Because GCDH activity is central to the catabolism of lysine and tryptophan, glutaric acid (GA) and related metabolites accumulate in the tissues and fluids of affected patients. Untreated patients are prone to develop severe striatal degeneration and irreversible movement disorders after the acute encephalopathic crises that occur early during development, between the ages of 3 and 36 months [3], [4]. Previous investigations have shown that early diagnosis and treatment can improve the prognosis of patients with GA1 significantly, but the outcomes can still vary, even among patients who follow their therapeutic regimens closely [3], [5], [6]. Despite extensive experimental work, the mechanisms underlying the development of striatal lesions remain unclear. This limits the design of appropriate therapeutic approaches [7]–[9].

In previous studies, several *in vitro* and *in vivo* model systems have been used to investigate the pathogenesis of neurodegeneration. *In vitro* studies have mainly focused on the neurotoxicity of GA and related metabolites, but have not considered the interactions among related metabolites [10]–[12]. Animal models include *Rousettus aegypticus*, chemical animal models (created using intracerebroventricular, intrastriatal, and subcutaneous administration of GA in rats), knock-out (KO) mouse models, and diet-induced KO mouse models [7], [13]–[16]. These models have provided insight into individual pathological mechanisms, but the results have not been consistent across different models and human patients. At present, innovative *in vitro* and *in vivo* models mimicking the metabolic impairment in GA1 patients are needed for a better comprehension of the mechanisms involved in the neuropathogenesis in GA1 [7].

Short hairpin RNA (shRNA) and small interfering RNA (siRNA) are used to specifically suppress the transcription of specific target genes [17]. However, shRNA and siRNA are difficult to transduce into neurons. Lentivirus-mediated shRNA can introduce genetic material into neurons and integrate into the host genome readily, producing stable and persistent suppression of target gene both *in vitro* and *in vivo* [18]. Lentiviral vectors have become the most widely used vectors for biological research and functional genomics, and shown great promise for clinical applications [19]–[22]. This technology has been used in the investigation of Huntington’s disease, which is a hereditary neurodegenerative disorder similar to GA1 [23]–[25]. The lentivirus system thus provides a new investigative perspective regarding the exploration of mechanisms involved in the neuropathogenesis in GA1.

In this study, we used lentivirus-mediated shRNA to suppress the expression of GCDH gene in rat striatal neurons. These neurons were cultured with a high concentration of lysine to imitate the hypermetabolic state of GA1 patients during acute encephalopathic crisis. We found that suppression of the GCDH gene and excessive intake of lysine induced apoptosis in rat striatal neurons. Our results suggest that lentivirus-mediated targeted suppression of GCDH gene might be a more useful means of determining the mechanism underlying GA1-induced striatal degeneration and whether the observed cell death is partially caspase-dependent.

## Materials and Methods

### Ethics

This study was carried out in strict accordance with the Guide for the Care and Use of Laboratory Animals issued by the National Institutes of Health. The protocol was approved by the Committee on the Ethics of Animal Experiments of Tongji Medical College (Permit Number: 2011-S248). Every effort was made to minimize the animals’ suffering.

### Culture and Identification of Primary Striatal Neurons

Neonatal rats (Sprague-Dawley) were killed by decapitation on postnatal day 1. All animals were purchased from the Experimental Animal Center of Tongji Medical College. Primary striatal neurons were cultured using a slightly modified version of a procedure described in a previous study [26]. Briefly, striatum tissues were cut into 1 mm^3^ fragments and incubated with 0.125% trypsin (Sigma) for 15 min at 37°C. Neurons were plated at a density of 5×10^5^ cells/well onto 6-well plates coated with 0.1 mg/ml poly-L-lysine (Sigma). After 4 h, plating medium (80% Dulbecco’s modified Eagle’s medium-high glucose medium (Hyclone), 10% fetal bovine serum (Gibco), and 2 mmol/L glutamine (Sigma)) was replaced with maintenance medium (98% neurobasal A medium (Gibco), 2% B27 (Gibco, containing serum-free supplements for growth and long-term viability of neurons [27]), 0.5 mmol/L glutamine).

Cultures were washed with PBS (0.01 mol/L phosphate buffer solution) before and after fixation with 4% paraformaldehyde for 30 min. The fixed cultures were then permeabilized with 0.3% TritonX-100 in PBS for 30 min, washed in PBS, and incubated overnight with primary antibody at 4°C. The primary antibody, a polyclonal rabbit anti-rat microtubule-associated protein antibody (MAP2, Proteintech Group, Inc. China), was diluted to 1∶100 in 2% goat serum-PBS containing 3% bovine serum albumin and 0.3% Triton X-100. Cells were then incubated with Texas Red-conjugated secondary antibody (Jackson, Inc. China, 1∶250) for 2 h. For nuclear staining, these cells were washed and incubated with Hoechst33342 (5 µg/ml, Sigma) for 5 min. Stained cells were visualized under a fluorescence microscope (Olympus BX51 AX-70, Japan). Images were analyzed using Image-Pro Plus 6.0 (Media Cybernetics, Bethesda, MD, U.S.).

Cultures were harvested and washed in PBS (mixed with 1% bovine serum albumin), and then cell density was maintained at 10^6^ cells/ml. The cells were incubated with primary antibody (MAP2, diluted to 1∶100) for 20 min. Then the cells were washed in PBS again and stained with secondary antibody (diluted to 1∶250) in the dark for 30 min. After staining, cells were washed again and analyzed using a flow cytometry analyzer (BD Biosciences).

### Lentiviral shRNA Vector Construction and Transfection

Three target shRNAs against rat GCDH gene (Gene Bank accession NM_001108896.1) were designed as follows: shRNA#1∶5′-GGAGCAGCGACAGAAGTAT-3′, shRNA#2∶5′-GGACAAGGCTACTCCAGAA-3′, and shRNA#3∶5′-GGGACATTGTATATGAGAT-3′. Oligonucleotides encoding shRNA sequences and one negative control sequence (5′-TTCTCCGAACGTGTCACGT-3′, which showed no significant homology to any mouse or human gene [28]) were synthesized and annealed into double strands. Double-stranded DNAs were inserted into Hpa1/Xho1 restriction sites of lentiviral frame plamids (Figure S1, pFU-GW-RNAi, encoding green fluorescent protein (GFP), the lentiviral frame plasmid was supplied by Genechem Co. Shanghai, China). They were then transformed into E.coli and positive recombinant clones were selected by using PCR, using the primers 5′-GCCCCGGTTAATTTGCATAT-3′ and 5′-GAGGCCAGATCTTGGGTG-3′. The conditions for the PCR were denaturation at 94°C for 30 sec, then 94°C for 30 sec, 55°C for 30 sec and 72°C for 30 sec, for 35 cycles, and extension at 72°C for 6 min. The products were then electrophoresed on a 1.5% agarose gel containing ethidium bromide. The length of positive clones containing shRNA was 343 bp, and the length of blank clones was 299 bp. Recombinant non-integrative lentiviral vectors were produced by co-transfecting 293T cells with the lentivirus expression plasmid and packaging plasmid (pHelper 1.0 including gag/pol and pHelper 2.0 including VSVG) using Lipofectamine 2000 (Invitrogen) [29]. Forty-eight hours later, the supernatants were collected and concentrated. After transfection, the viral titer was determined by counting GFP-positive cells. The viral titer was then diluted to 10^8^ TU/ml. DNA sequencing results revealed that the RNA interference sequence targeting the GCDH gene was successfully inserted into the lentiviral vector.

Transfection efficiency was determined using the negative control (NC) lentivirus. After 10 days of culture, cells were infected at various multiplicities of infection (MOI: 1, 10, 20). Then, 72 h after infection, the transduction efficiency was observed under a fluorescent microscope (Figure S2). The best MOI was found to be 10. Then digestion was performed and single cell suspension was prepared (2×10^5^ cells in 200 µl PBS). The GFP intensity was determined by flow cytometry. When MOI was at 10 (Figure S2), the transfection efficiency was 96.5±2.3% based on flow cytometry results.

Cells were divided into three groups: a control group (uninfected), a NC group (transfected with negative control virus), and a lentivirus-shRNA group (transfected with target shRNAs lentiviral vectors). The lentivirus-shRNA group was divided into three subgroups based on shRNA sequence: lentivirus-shRNA#1, lentivirus-shRNA#2, and lentivirus-shRNA#3. Interference efficiency was detected using RT-PCR and Western blotting.

### MTT Assay

Primary striatal neurons were seeded into 96-well plates at a density of 5×10^4^ cells/well. Neurons were incubated with 0 mmol/L, 5 mmol/L, 10 mmol/L, 15 mmol/L, or 20 mmol/L lysine (Sigma) for 24 h. Then 3-(4, 5)-dimethylthiahiazo-3, 5-diphenytetrazoliumromide (MTT, Sigma) was added to these wells and incubated (at a concentration of 500 mg/L) for another 4 h. The medium was then removed, and 150 µL dimethyl sulfoxide was added and concussed for 10 min. Another three wells containing no cells were filled with 100 µL medium. These served as blank controls. Opacity density (OD) was measured at 570 nm using a spectrophotometer. Cell viability (%) = (OD of cells with different treatments – OD of blank control)/(OD of cells with no treatment – OD of blank control) ×100. We did not detect any observable differences in survival between cells exposed to 0 and 10 mmol/L lysine (Table S1). A lysine concentration of more than 15 mmol/L was found to be toxic to the neurons. Three groups of cells (control, NC, lentivirus-shRNA) were incubated with 0 mmol/L, 5 mmol/L, or 10 mmol/L lysine for 24 h. Cell viability was assessed using MTT assay.

### Hoechst Staining Assay

Cultures were stained with Hoechst 33342 (10 µg/ml, Sigma) for 10 min. Changes in nuclear morphology were observed using fluorescent microscopy (350 nm stimulation and 460 nm emission). The relative number of Hoechst-positive nuclei per visual field (minimum of 10 fields) was determined.

### Annexin V-PE/7-AAD Staining

Cells were trypsinized and washed with serum-containing medium. The samples (5×10^5^ cells) were centrifuged for 5 minutes at 400×g and the supernatant was discarded. The cells were then stained using an Annexin V-PE/7-AAD apoptosis kit (MultiSciences Biotech Co, Ltd) in accordance with the manufacturer’s instructions. The number of apoptotic cells was detected and analyzed using flow cytometry.

### Determination of Mitochondrial Membrane Potential (MPP)

Cells in 3.5 cm culture dishes (5×10^4^ cells/dish) were washed three times with Tyrode’s buffer and then incubated with tetramethylrhodamine methyl ester (TMRM, 20 nmol/L, Sigma) in the dark at room temperature. After 45 min, the cultures were washed 4 times with Tyrode’s buffer and mounted on the stage of a confocal laser scanning microscope (LSM 510, Carl Zeiss Inc.). All procedures were performed as described previously [30]. We used a region of interest tool (ROI) from the LSM program to select the areas and measure TMRM fluorescence intensities. We calculated the average fluorescence intensities of all ROIs and the background fluorescence intensities of the regions next to the cells. After subtracting background intensity, we normalized the TMRM fluorescence intensities using the following formula (△F =  (F_0_–F)/F_0_; where F_0_ =  fluorescence intensity in the NC group, F =  fluorescence intensity in other groups).

Cells were harvested as described in the Annexin V-PE/7-AAD staining section. The cells were resuspended with PBS and incubated with TMRM (20 nmol/L) in the dark at room temperature. After 30 min, the cells were rewashed and suspended with 200 µl PBS. Then the TMRM signal was analyzed in the FL2 channel of a flow cytometry analyzer [31].

### Real-time Reverse Transcription Polymerase Chain Reaction (RT-PCR)

Primary striatal neurons were seeded in 6-well plates at a density of 5×10^5^ cells/well. Total RNA was extracted using Trizol (Invitrogen). Complementary DNA was synthesized in accordance with the manufacturers protocol (Toyobo, Japan). Real-time PCR amplification was performed on an ABI PRISM 7500 cycler with SYBR reagent (Toyobo, Japan). The thermal cycling conditions were set as given in the instructions included with the cycler, and the annealing temperature was 60°C. The sense primer 5′- GAAAGCCCTGGACATCG -3′ and the antisense primer 5′- CAACCGTGAATGCCTGA -3′ were used for amplification of GCDH (designed by Primer 5.0, synthesized by Invitrogen, China). Quantitative normalization of cDNA in each sample was performed using rat housekeeping gene glyceraldehyde-3-phosphate dehydrogenase (GAPDH, sense primer, 5′- TTCAACGGCACAGTCAAGG -3′; antisense primer, 5′- CTCAGCACCAGCATCACC -3′) as an internal control to determine the uniformity of the template RNA for all specimens. For each sample, GCDH expression was derived from the ratio of its own expression to GAPDH expression using the following formula: relative expression  = 2^− (△Ct sample−△Ct control)^, △Ct = Ct_GCDH_−Ct_GAPDH_.

### Western Blotting

Cells were plated in 6-well plates at a density of 5×10^5^ cells/well and lysed using cell lysis buffer (Beyotime, China) and phenylmethylsulfonyl fluoride (PMSF, Sigma). Protein extracts were quantified using a BCA protein assay kit (Beyotime, China). Denatured protein samples (40 µg/lane) were separated using 10% sodium dodecyl sulfate polyacrylamide and transferred onto polyvinylidene difluoride membranes. The membranes were sealed with skim milk powder and incubated with primary antibodies for 2 h at room temperature. Primary antibodies for GCDH (Proteintech Group, Inc. China) were diluted to 1∶300 in 5% skim milk powder with 0.2% PBS-Tween 20. Primary antibodies against β-actin were diluted to 1∶1000, and antibodies against caspase 3 (Santa Cruz Biotechnology, Inc.), caspase 8, and caspase 9 (Cell Signaling Technology, Inc.) were diluted to 1∶500. The membranes were washed three times (5 min/wash) using Tris-buffered saline with Tween-20, (pH 8.0). These were then incubated with horseradish-peroxidase-conjugated secondary antibodies (Jackson ImmunoResearch, PA, U.S., 1∶3000) for 2 h at room temperature. The blots were washed three times with PBS-Tween 20 and developed with enhanced chemiluminescence substrate (Amersham Pharmacia Biotech, Piscataway, NJ, U.S.). Protein bands were imaged using a gel image processing system (UVP Labworks, Upland, CA, U.S.) and quantified by densitometry (Quantity One). β-actin was used as a protein loading control.

### Statistical Analysis

All experiments were performed in triplicate. Data are presented as mean ± standard deviation. Statistical analysis was performed using SPSS17.0. Differences between two groups were compared using the Student’s t test, and the comparison among more than two groups was performed via analysis of variance (ANOVA) and the Student-Newman-Keuls test. *P*<0.05 was considered statistically significant.

## Results

### Assessment of the Neuron Purity

**Figure 1:**
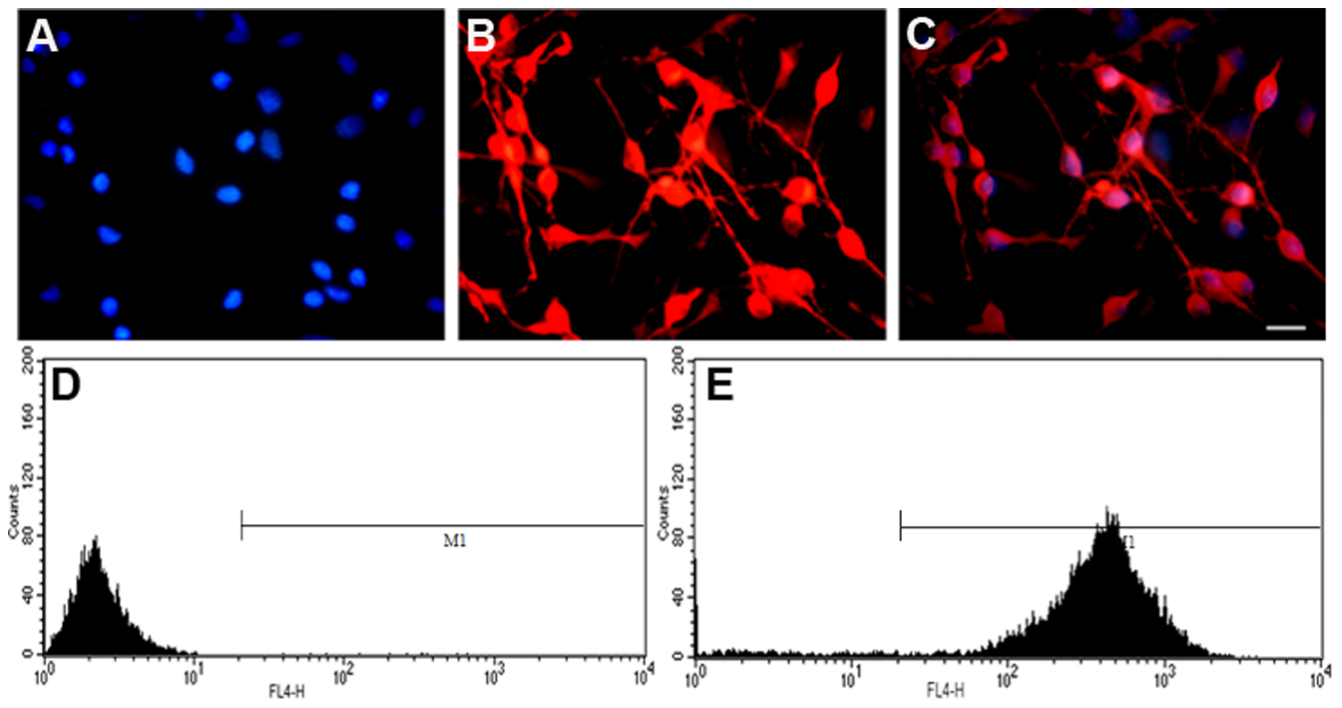
Assessment of neuronal purity. Immunofluorescence staining reveals the proportion of neurons in living cells to be 92.4±1.6%. Flow cytometry showed the proportion of neurons in living cells to be 94.3±2.5%. A: All nuclei were stained blue by Hoechst33342. B: All neuronal bodies and dendrites were labeled red by Texas Red. C: A merged image showing Hoechst33342 staining and Texas Red labeling. Scale bars: 20 µm. D: Cells without staining were analyzed by flow cytometry. E: Stained cells were analyzed by flow cytometry.

MAP2, which is mainly distributed in the neuronal bodies and dendrites, is widely used in the identification of nerve cells. In this study, all nuclei were stained blue with Hoechst33342, and all neuronal bodies and dendrites were stained red with Texas Red. In cultured isolated neurons, 92.4±1.6% of living cells was found to be MAP2-positive using immunofluorescence, and 94.3±2.5% of cells was found to be MAP2-positive using flow cytometry (Fig. 1).

### Assessment of Interference Efficiency

**Figure 2:**
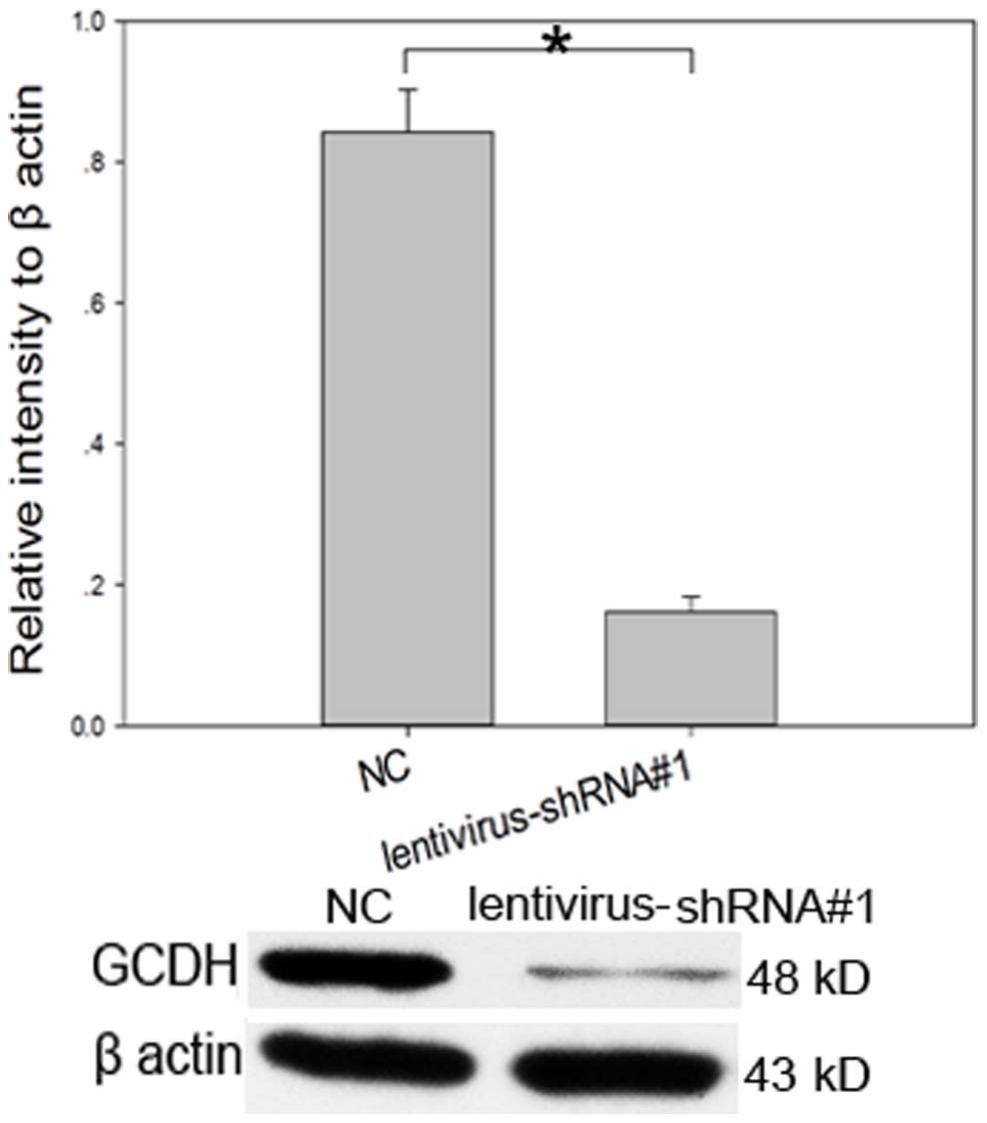
Efficiency of lentivirus-shRNA#1 interference as detected by Western blotting. GCDH expression in rat striatal neurons 72 h after infection with lentivirus. The lentivirus-shRNA#1 reduced the level of protein expression in GCDH by as much as 80.78% relative to the negative control lentivirus. *P<0.05.

The mRNA levels of GCDH as measured by RT-PCR in the lentivirus-shRNA#1, 2, and 3 subgroups were reduced by 63.4%, 54.2%, and 61.0%, respectively (Table 1). In view of the fact that many patients have some residual GCDH activity, which can reach 40% of normal levels and the fact that no association has been found between residual activity and clinical phenotype, suppression of the expression of GCDH gene by as much as 60% is sufficient for investigations of the mechanism of GA1 [32]–[35]. We assessed the efficiency of lentivirus-shRNA#1 interference using Western blotting. The level of protein expression was reduced by 80.78% (Fig. 2). The results suggest that the use of lentivirus-shRNA#1 is appropriate for the following experiments.

**Table 1:** Relative expression levels of GCDH in different groups as detected by RT-PCR.

	△Ct(GCDH-GAPDH)	Relative expression
**control**	7.719±0.1233	1.129
**NC**	7.893±0.2401	1
**Lentivirus-shRNA#1**	9.343±0.0306*	0.366
**Lentivirus-shRNA#2**	9.020±0.0100*	0.458
**Lentivirus-shRNA#3**	9.253±0.0153*	0.390

* *P<*0.05 *vs.* NC group. There were no significant differences between the control and NC group with respect to the level of GCDH mRNA level. The mRNA levels of GCDH in lentivirus-shRNA#1, 2, and 3 subgroups were reduced by 63.4%, 54.2%, and 61.0%, respectively.

### Neuronal Viability after Treatment with Lentivirus-shRNA#1 and Lysine

A concentration gradient (0–20 mmol/L) of lysine incubated with the cells reveal that cell survival was not affected by lysine at concentrations below 10 mmol/L (Table S1). When the concentration of lysine was no greater than 5 mmol/L, there was no significant difference in viability between the NC and control group (Table 2), suggesting that the defective virus and low doses of lysine (≤5 mmol/L) were nontoxic to cells. When lysine levels were higher than 10 mmol/L, the viability of neurons infected with NC lentivirus and lentivirus-shRNA#1 were reduced to varying degrees. When cells were treated with 5 mmol/L lysine, lentivirus-shRNA#1 reduced neuronal survival by 60.94% relative to cells transduced with the NC lentivirus. Lentivirus-shRNA#1 alone reduced neuronal survival by 24.05% relative to NC lentivirus. In GA1 patients, neurons gradually and progressively degenerate. Hypermetabolic states can develop, exacerbating degeneration [3]. In our study, GCDH-deficient neurons partially degenerated and 5 mmol/L lysine exacerbated this degeneration. In view of the fact that high-lysine diets do not induce neurodegeneration in normal children, 5 mmol/L lysine was used in the following experiments.

**Table 2:** OD in the detection of neuron viability by MTT assay.

	OD	Viability rate (%)
**0 mmol/L lysine**	0.510±0.0189	100%
**NC**	0.485±0.0085	92.67%
**NC+5 mM lysine**	0.476±0.0100	90.21%
**NC+10 mM lysine**	0.461±0.0097	85.83%*
**Lentivirus-shRNA**	0.403±0.0067	68.62%*
**Lentivirus-shRNA+5 mM** **lysine**	0.268±0.0070	29.27%*
**Lentivirus-shRNA+10 mM lysine**	0.245±0.0172	22.48%*

Viability rate (%)  = (OD_m_−OD_blank_)/(OD_0_−OD_blank_); OD_m:_ The OD of each sample; OD_0_: The OD of neurons with 0 mmol/L lysine group. OD_blank_: The OD of the blank control (0.169±0.0252).

* *P<*0.05 *vs.* neurons with 0 mmol/L lysine group.

**Figure 3:**
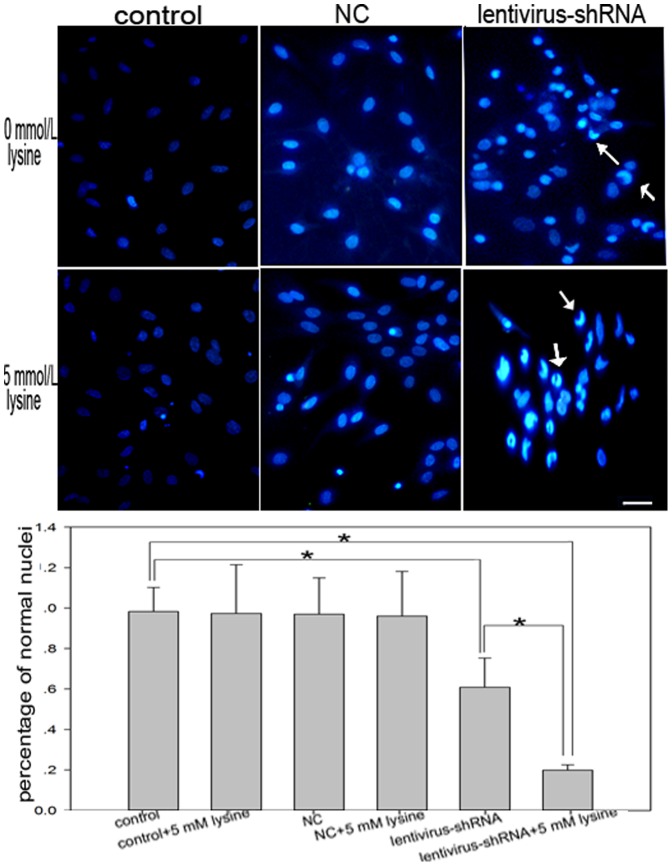
Hoechst 33342 staining of apoptotic neurons. The effects of GCDH knockdown and excess lysine on the nuclear morphological changes in rat neurons. Nuclei in uninfected neurons and neurons infected with negative control lentivirus were lightly stained blue. Apoptotic nuclei were deeply stained blue, and appeared dense and fragmented (marked with arrows). Scale bars: 20 µm. The histogram represents the percentage of apoptotic cells. *P<0.05.

As shown in Figure 3, nuclei were lightly stained blue in the NC and control groups, and there was no significant apoptosis in either group. Lentivirus-shRNA#1 increased the rate of neuronal apoptosis by 36.22% relative to NC lentivirus. When cells were treated with 5 mmol/L lysine, lentivirus-shRNA#1 increased the level of neuron apoptosis by as much as 76.21% relative to NC lentivirus. These results were consistent with the MTT assay results.

**Figure 4:**
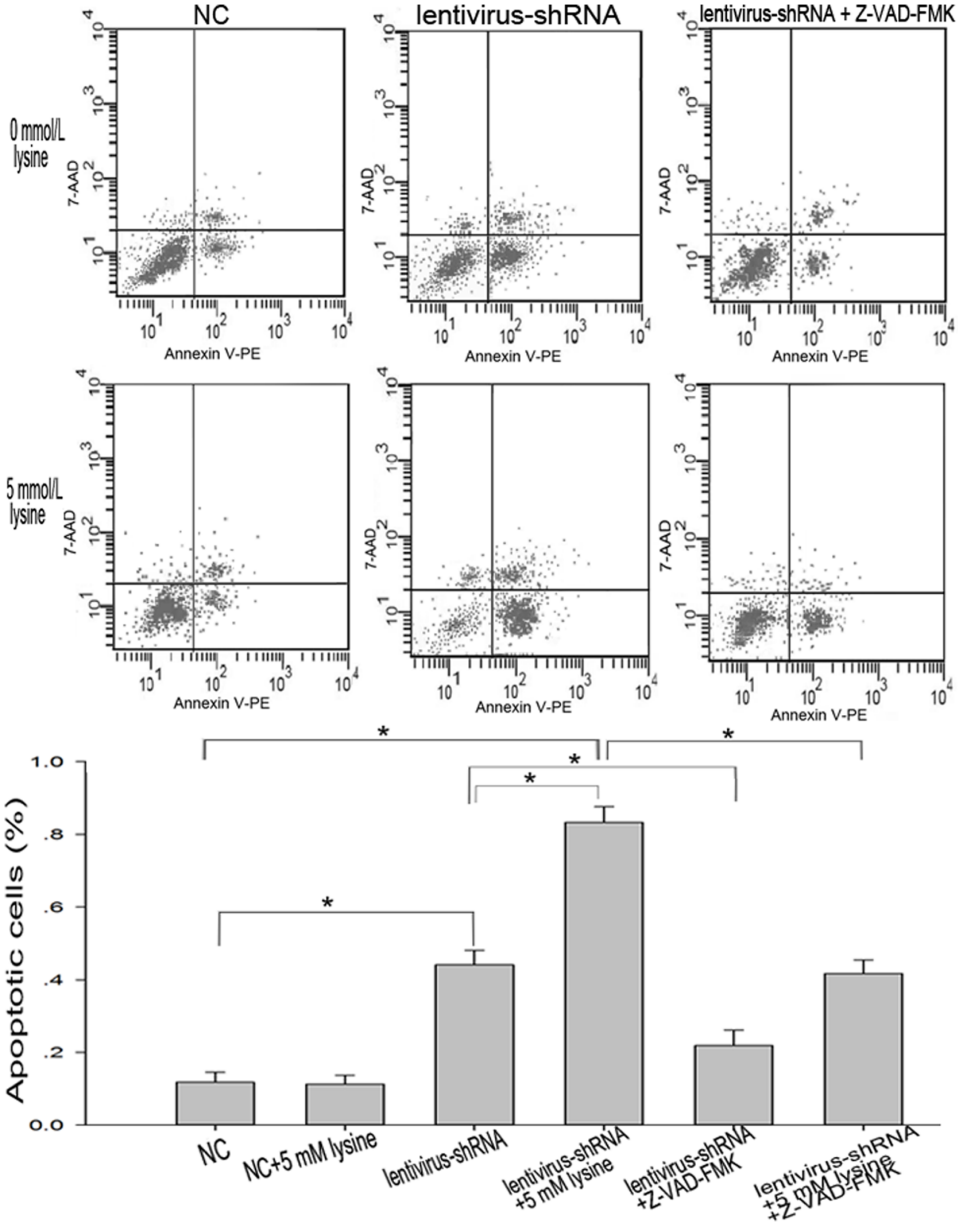
Detection of apoptosis using flow cytometry. Cells were assayed for apoptosis using Annexin V-PE/7-AAD staining with flow cytometry. Cells were grouped and treated as shown to quantify the apoptosis induced by GCDH knockdown and increased lysine. Lentivirus-shRNA#1 induced apoptosis, and 5 mmol/L lysine increased the rate of apoptosis to a significantly greater extent. Z-VAD-FMK, a pan-caspase inhibitor, blocked the apoptosis induced by lentivirus-shRNA and increased lysine to a great extent. *P<0.05.

In order to confirm the effects of lentivirus-shRNA#1 and increased lysine level on neurons, we quantified the number of apoptotic cells using Annexin V-PE/7-AAD staining and flow cytometry (Figure 4). Because there was no significant difference in the viability between the NC and control group that were either exposed to additional 5 mmol/L lysine or not, we quantified apoptosis relative to the NC lentivirus group. Increased lysine did not change the apoptotic cell fraction in neurons infected with NC lentivirus. The apoptotic cell fraction was significantly higher in the lentivirus-shRNA#1 group: 44.13% in cells not exposed to lysine and 83.35% in cells exposed to 5 mmol/L lysine. These suggest that GCDH downregulation through lentivirus-shRNA#1 induced neuronal apoptosis and increased lysine level enhanced this apoptosis.

### Assessment of MPP

**Figure 5:**
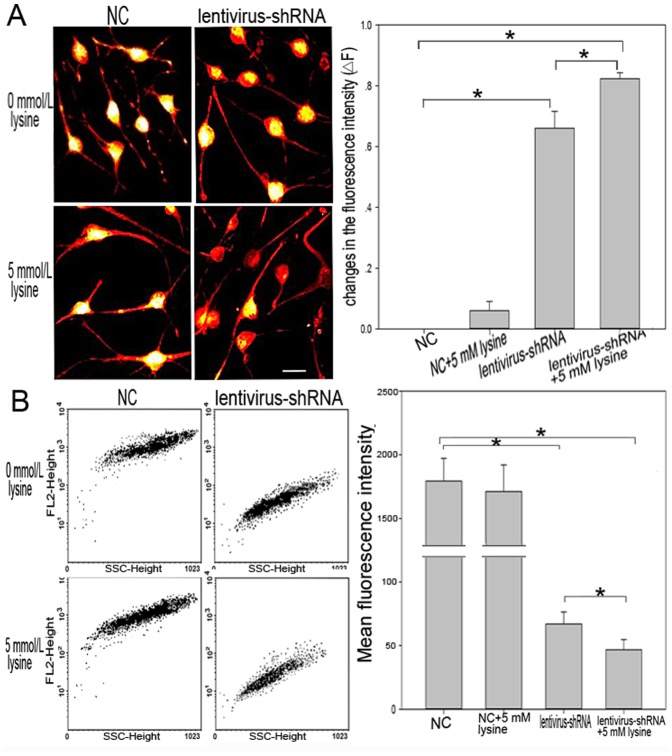
Assessment of MPP in rat striatal neurons. A: Fluorescence images of rat striatal neurons incubated with TMRM. Lentivirus-shRNA#1 leads to mitochondrial depolarization and loss of fluorescence intensity. The loss of TMRM fluorescence from the mitochondrial regions indicates the collapse of MPP upon lentivirus-shRNA#1 and lysine treatment. Scale bars: 20 µm. The histogram shows the quantitative representation of changes in the fluorescence intensity of TMRM upon different treatments. △F = (F_0_–F)/F_0_; F_0_: TMRM fluorescence intensity in the lysine-free NC group; F: TMRM fluorescence intensity in other groups. *P<0.05. B: MPP was assessed using flow cytometry. Abscissa represents SSC-height (side scatter height), ordinate intensity of fluorescence. The histogram shows the changes in mean fluorescence intensity of all the cells. *P<0.05.

The collapse of MPP is the critical first step in apoptosis [36]. Here, we report the differences in MPP status between the experimental and NC groups. TMRM fluorescence intensity was proportional to the level of MPP, as shown in Figure 5. Lentivirus-shRNA#1 was found to markedly decrease MPP regardless of the presence of lysine, and 5 mmol/L lysine enhanced this decrease. Quantification performed using flow cytometry was consistent with the results of LSM.

### Expression of Apoptosis-related Proteins

**Figure 6:**
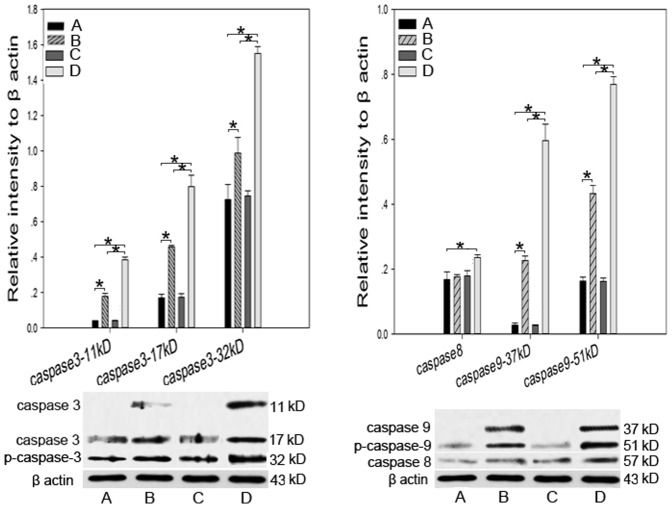
Protein expression of caspases 3, 8, and 9. (A) NC; (B) lentivirus-shRNA#1; (C) NC +5 mmol/L lysine; and (D) lentivirus-shRNA#1+5 mmol/L lysine. *P<0.05. The protein levels of caspases 3 and 9 were significantly upregulated by lentivirus-shRNA#1, and this upregulation was intensified by 5 mmol/L lysine. Neither lentivirus-shRNA#1 nor 5 mmol/L lysine changed the expression of caspase 8 alone. Exposure to both conditions increased the protein level of caspase 8.

Because GA-related metabolites can induce apoptosis in neurons, we evaluated the expression of apoptosis-related proteins using Western blotting (Figure 6). The protein levels of caspases 3 and 9 were significantly upregulated by lentivirus-shRNA#1. The combination of lysine and lentivirus-shRNA#1 intensified the upregulation of caspases 3 and 9. Neither lentivirus-shRNA#1 nor 5 mmol/L lysine alone changed the level of caspase 8 expression, but exposure to both increased the protein level of caspase 8.

### Effects of Caspase Inhibitor on Apoptosis Induced by GA-related Metabolites

To confirm the importance of caspase-dependent processes in apoptosis induced by GA-related metabolites, we included the pan-caspase inhibitor benzyloxy-carbonyl-Val-Ala-Asp(OMe)-fluoromethylketone (Z-VAD-FMK, MPBio U.S.) in our experiments. This compound did not affect the survival of rat neurons when used at 100 µmol/L [37]. Z-VAD-FMK was added to the medium 1 h prior to lentiviral infection. This blocked the suppressive effective of the metabolites on the viability of rat neurons to a significant extent, as indicated by flow cytometry (Figure 4). With Z-VAD-FMK pretreatment, the apoptotic cell fraction in cells infected with lentivirus-shRNA#1 decreased to 21.87% in cells not exposed to lysine and 41.66% in cells exposed to 5 mmol/L lysine. This confirmed that lysine-related metabolites induced apoptosis in a partially caspase-dependent manner.

## Discussion

Our constructed lentiviral vector displayed high infection efficiency in primary striatal neurons and remarkably suppressed the expression of GCDH gene. GCDH is located in the mitochondrial matrix. Lysine is transported into the mitochondria and degraded into glutaryl-CoA. When GCDH levels is low, glutaryl-CoA cannot be catalyzed to crotonyl-CoA, and the generation of GA, 3-hydroxyglutaric acid (3-OHGA), and glutarylcarnitine are all increased [36], [38]. About 10–20% of GA1 patients are regarded as insidious-onset or late-onset. These patients do not experience any documented encephalopathic crises [3], [39], [40]. This means that GA1 patients still suffer from neural degeneration even when no observable hypermetabolic events take place, which can exacerbate degeneration. In this study, the GCDH-deficient striatal neurons caused by lentivirus-shRNA#1 were found to be partly apoptotic.

Acute encephalopathic crises are often precipitated by events such as surgical intervention, febrile illness, and vaccination. Under hypermetabolic conditions, hypoglycemia stimulates the conversion of energy substrates in the brain to ketogenic amino acids and ketone bodies. The increased utilization of lysine in the brains of GA1 patients can enhance glutarate accumulation and inhibit the Krebs cycle [41]. This in turn inhibits gluconeogenesis resulting in hypoglycemia. These series of events constitute a vicious cycle. Low-lysine and high-arginine diets have been widely used in GA1 therapy. In most proteins, lysine is more abundant than tryptophan. Lysine breakdown increases substantially during catabolic crisis [7]. Approximately 90% of untreated GA1 patients develop neurodegenerative disease during brain development after acute encephalopathic crisis. In our study, excessive lysine intake (higher levels of lysine-related metabolites) promoted the apoptosis induced by lentivirus-shRNA. We speculate that 5 mmol/L lysine may simulate catabolic crisis in this GA1 model.

Previous *in vitro* models have focused mainly on organotypic slices or on neuronal cells incubated with GA, 3-OHGA, or other related metabolites. They have facilitated the development of a considerable number of hypotheses regarding neuropathogenesis, but many of these hypotheses are controversial. Some have shown GA and 3-OHGA to act as direct or indirect neurotoxins, while others have indicated no neurotoxicity. It has been suggested that astrocytes may protect neurons from the excitotoxic damage caused by 3-OHGA [41]. Neuronal cultures have been shown to be more vulnerable to 3-OHGA than mixed-cell cultures [42]. However, experiments have also provided evidence that reactive glial cells may at least partially underlie the neuropathology of GA1 [43]. Other experiments have shown that GA does not induce neuronal death in the absence of astrocytes and that neonatal astrocyte damage is sufficient to trigger progressive striatal degeneration. In this case, neuronal death appeared several days after GA treatment and increased progressively [13]. However, in GA1 patients, neuronal loss occurs shortly after the encephalopathical crisis and does not progress [44]. Because existing *in vitro* models have produced profoundly conflicting results, further research should be performed and a new, more complex model should be developed.

Many factors limited these previous studies. Firstly, in GA1 patients, GA and other metabolites are generated within the cell and mitochondria, and intracellular GA accumulation may cause direct mitochondrial toxicity within neurons. Furthermore, GA-related metabolites have never been examined for its impact on cell-membrane receptors. This is the limitation in the described *in vitro* models, conducted in organotypic slices or neuronal cells incubated with GA-related metabolites. Secondly, the intracellular levels of GA or 3-OHGA are unknown, and they could be present in cells at an order of magnitude higher than those used in previous *in vitro* models. Thirdly, the interaction among related metabolites was not considered in previous *in vitro* models.

Since experiments have demonstrated that the expression of GCDH is restricted to neurons in normal mouse brains [45], we focused on GCDH-deficient striatal neurons. In this novel GA1 model established using lentivirus mediated shRNA, GCDH-deficient striatal neurons were found to undergo apoptosis. All GA-related metabolites were generated at the mitochondria, and they acted either intracellularly or extracellularly. All metabolites, even those related to carnitine-deficiency, were found to interact with each other and collectively influence the viability of striatal neurons.

Pieces of evidence have demonstrated that intracerebral de novo synthesis of GA and other metabolites and subsequent limited transportation across the blood-brain barrier may be involved in neuronal damage observed in GA1. This observation has inspired the design of KO mouse models [46]–[49]. The biochemical phenotypes of these mice are similar to those of GA1 patients, but these mice do not develop striatal injury spontaneously [15]. KO mice fed with a high-lysine diet develop severe neuropathology, similar to that of GA1 patients, but the findings regarding the pathologic role of dicarboxylic acid in their brains have not been consistent [16], [50]. These differences may be due to intrinsic differences between the striata of mice and of humans. Because the genome of mice is similar to those of humans and because mice are easy to handle, mice are widely used in gene knockout experiments. Rat is the traditional animal of choice in investigating the central nervous system of humans, since it offers considerable advantages over mouse and is more similar to human than mouse with respect to the central nervous system [51]–[54]. Lentivirus-shRNA can integrate into the genomes of neurons to produce stable, long-term silencing [55], [56]. Therefore, intrastriatal administration of lentivirus-shRNA in neonatal rats may be suitable for the establishment of a novel *in vivo* model. Moreover, this model may be less expensive and easier to handle than the KO mouse model.

Increasing evidence shows that mitochondrial dysfunction is involved in the pathology of various organic acidemias and neurodegeneration [57], [58]. In the present study, both LSM and flow cytometry results revealed that lentivirus-shRNA#1 markedly decreased MPP levels, and that 5 mmol/L lysine enhanced this decrease. These results indicate that mitochondrial dysfunction is involved in striatal neurodegeneration in GA1. Several lines of evidence have suggested that mitochondrial disruption is involved in the brain injuries sustained by GA1 patients [45]. Other experiments have shown that bioenergetic impairment is involved in the neurodegenerative changes associated with GA1 and demonstrated that mitochondrial disruption plays an important role in striatal neurodegeneration in GA1 [59]–[61].

The collapse of MPP is the critical first step in apoptosis. Caspase 8 is an important initiator of the extrinsic pathway. Caspase 9 is an important initiator of the intrinsic pathway, and caspase 3 is the major executor in cell apoptosis. A great deal of evidence has shown that caspases contribute to neurodegeneration in Alzheimer’s disease [62]. However, investigation into the correlation between caspase activity and neurodegeneration in GA1 has been limited. In this study, the protein levels of caspase 3, 8, and 9 were detected and used to identify the apoptotic pathways most likely to be involved in GA1. The levels of caspases 3 and 9 (precursors and cleaved fragments of both) were higher in cells infected with lentivirus-shRNA#1 than in the NC group. In these cells, co-treatment with 5 mmol/L lysine increased the level of caspase 8. Pretreatment with Z-VAD-FMK decreased the number of lentivirus-shRNA#1-infected cells that are apoptotic, which suggests that the apoptosis induced by lysine-related metabolites might be partially caspase-dependent.

In conclusion, we successfully established a novel cell model of GA1 using lentivirus-mediated shRNA to GCDH and excessive intake of lysine. Intrastriatal administration of lentivirus-shRNA in rats may offer another appropriate *in vivo* model for the study of GA1. This study provides evidence that GA1-triggered apoptosis in neurons is partially caspase-dependent. The specific details of the mechanisms and molecular players involved in this apoptosis merit further research. Indeed, many novel mitochondrial targets for neuroprotection have been identified providing more alternatives in addressing GA1 [63].

## Supporting Information

Figure S1 **The diagram of pFU-GW-siRNA vector.** CMV/LTR:913-2415, U6 promoter: 2600–2915, Polylinker: 2916–2987, Ubiqutin Promoter:2955–4140, EGFP:4234–4953, LTR:5721–6293, Polylinker: Hpa I, Xho I. Polylinker: **GTTAAC** GCGCGGTGACC **CTCGAG** **.**
(TIF)

Figure S2 **Neurons infected with lentivirus.** Neurons were infected with negative control lentivirus at various MOI (1, 10, 20). Fluorescence images showed the best MOI to be 10. A: At MOI = 1, there was no fluorescence. B: At MOI = 10, more than 90% cells were green and showed normal morphology. C: At MOI = 20, nearly all the cells were infected, but some cells exhibited swollen bodies and sparse neurites. Scale bars: 20 µm. Flow cytometry results reveal the transfection efficiency to be 96.5±2.3% when MOI is at 10. A: Uninfected neurons were analyzed by flow cytometry. D: At MOI = 10, cells were analyzed by flow cytometry.
(TIF)

Table S1 **OD in the detection of neuron viability by MTT assay.** Viability rate (%)  = (OD_m_-OD_blank_)/(OD_0_− OD_blank_); OD_m:_ The OD of each sample; OD_0_: The OD of neurons with 0 mmol/L lysine group. OD_blank_: The OD of the blank control (0.172±0.0297). *P<0.05 *vs*. neurons with 0 mmol/L lysine group.
(DOC)
