# Supplementary figures and images for: Effects of Targeted Suppression of Glutaryl-CoA Dehydrogenase by Lentivirus-Mediated shRNA and Excessive Intake of Lysine on Apoptosis in Rat Striatal Neurons
Source: PLoS One. 2013 May 2;8(5):e63084. doi: 10.1371/journal.pone.0063084 (PMC3642093; doi:10.1371/journal.pone.0063084)

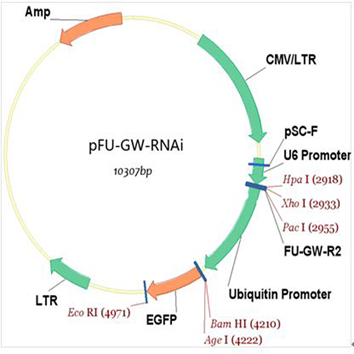

Supplement: Figure S1 — The diagram of pFU-GW-siRNA vector. CMV/LTR:913-2415, U6 promoter: 2600–2915, Polylinker: 2916–2987, Ubiqutin Promoter:2955–4140, EGFP:4234–4953, LTR:5721–6293, Polylinker: Hpa I, Xho I. Polylinker: GTTAAC GCGCGGTGACC CTCGAG . (TIF) [file pone.0063084.s001.tif]

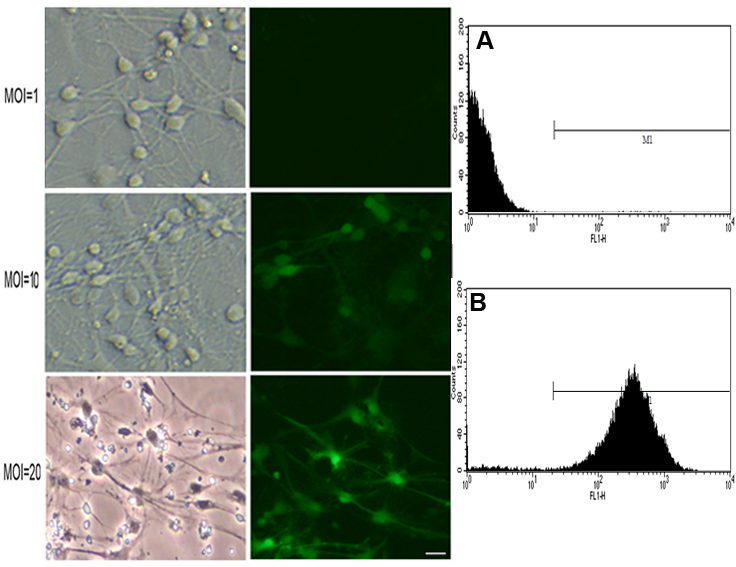

Supplement: Figure S2 — Neurons infected with lentivirus. Neurons were infected with negative control lentivirus at various MOI (1, 10, 20). Fluorescence images showed the best MOI to be 10. A: At MOI = 1, there was no fluorescence. B: At MOI = 10, more than 90% cells were green and showed normal morphology. C: At MOI = 20, nearly all the cells were infected, but some cells exhibited swollen bodies and sparse neurites. Scale bars: 20 µm. Flow cytometry results reveal the transfection efficiency to be 96.5±2.3% when MOI is at 10. A: Uninfected neurons were analyzed by flow cytometry. D: At MOI = 10, cells were analyzed by flow cytometry. (TIF) [file pone.0063084.s002.tif]
