# Supplementary material for: Effects of Targeted Suppression of Glutaryl-CoA Dehydrogenase by Lentivirus-Mediated shRNA and Excessive Intake of Lysine on Apoptosis in Rat Striatal Neurons
Source: PLoS One. 2013 May 2;8(5):e63084. doi: 10.1371/journal.pone.0063084 (PMC3642093; doi:10.1371/journal.pone.0063084)
Supplement: Table S1 — OD in the detection of neuron viability by MTT assay. Viability rate (%) = (ODm-ODblank)/(OD0− ODblank); ODm: The OD of each sample; OD0: The OD of neurons with 0 mmol/L lysine group. ODblank: The OD of the blank control (0.172±0.0297). *P<0.05 vs. neurons with 0 mmol/L lysine group. (DOC) [file pone.0063084.s003.doc]

**Supplemental Table 1. OD in the detection of neuron viability by MTT assay.**

| |  | **OD** | **Viability rare (%)** | | --- | --- | --- | | **0 mmol/L lysine** | 0.509±0.0018 | 100% | | **5 mmol/L lysine** | 0.506±0.0016 | 99.09% | | **10 mmol/L lysine** | 0.506±0.0015 | 99.10% | | **15 mmol/L lysine** | 0.457±0.0209 | 84.63%* | | **20 mmol/L lysine** | 0.326±0.0283 | 45.60%* | |
| --- | --- | --- | --- | --- | --- | --- | --- | --- | --- | --- | --- | --- | --- | --- | --- | --- | --- | --- |
